# Comparative genome analysis of the freshwater fungus *Filosporella fistucella* indicates potential for plant-litter degradation at cold temperatures

**DOI:** 10.1093/g3journal/jkad190

**Published:** 2023-08-24

**Authors:** Daniel Vasconcelos Rissi, Maham Ijaz, Christiane Baschien

**Affiliations:** Leibniz - Institute DSMZ, German Collection of Microorganisms and Cell Cultures, 38124 Braunschweig, Germany; Leibniz - Institute DSMZ, German Collection of Microorganisms and Cell Cultures, 38124 Braunschweig, Germany; Leibniz - Institute DSMZ, German Collection of Microorganisms and Cell Cultures, 38124 Braunschweig, Germany

**Keywords:** fungal genome, freshwater fungi, long-read sequencing, comparative genomics, cold-adapt proteins

## Abstract

Freshwater fungi play an important role in the decomposition of organic matter of leaf litter in rivers and streams. They also possess the necessary mechanisms to endure lower temperatures caused by habitat and weather variations. This includes the production of cold-active enzymes and antifreeze proteins. To better understand the physiological activities of freshwater fungi in their natural environment, different methods are being applied, and genome sequencing is one in the spotlight. In our study, we sequenced the first genome of the freshwater fungus *Filosporella fistucella* (45.7 Mb) and compared the genome with the evolutionary close-related species *Tricladium varicosporioides* (48.2 Mb). The genomes were annotated using the carbohydrate-active enzyme database where we then filtered for leaf-litter degradation-related enzymes (cellulase, hemicellulase, laccase, pectinase, cutinase, amylase, xylanase, and xyloglucanase). Those enzymes were analyzed for antifreeze properties using a machine-learning approach. We discovered that *F. fistucella* has more enzymes to participate in the breakdown of sugar, leaf, and wood than *T. varicosporioides* (855 and 719, respectively). *Filosporella fistucella* shows a larger set of enzymes capable of resisting cold temperatures than *T*. *varicosporioides* (75 and 66, respectively). Our findings indicate that in comparison with *T*. *varicosporioides*, *F*. *fistucella* has a greater capacity for aquatic growth, adaptability to freshwater environments, and resistance to low temperatures.

## Introduction

Aquatic habitats are classified according to their water flow, which can be artificial lentic, lotic artificial, or hybrid (Odum 1969). Lotic ecosystems, which include springs, rivers, and streams, are defined as waters that sustain a steady flow. Lentic ecosystems are static aquatic ecosystems such as lakes, ponds, and swamps (Kuehn 2016). Dams and reservoirs are examples of artificial or hybrid habitats (Jones and Pang 2011; Canto *et al.* 2022). The water flow is a determinant in the oxygenation of the lotic and lentic ecosystems and can influence biological processes and biodiversity. In the lotic environment, better water oxygenation contributes to its rich biodiversity (Penaluna *et al.* 2017; Canto *et al.* 2022).

Freshwater comprises <3% of the total water on earth and can include groundwater, streams, rivers, canals, and lakes, as well as amphibious habitats such as ditches, peatlands, and swamps (Webster 1992; Shearer *et al.* 2007; Barros and Seena 2022; Calabon *et al.* 2022). Aquatic fungi are investigated more in freshwater habitats with cool, clean, well-oxygenated, flowing water (Tsui *et al.* 2016).

The aquatic fungi are a polyphyletic group that comprehends over 3,800 species of fungi (Calabon *et al.* 2022) that have at least one life cycle completed in water, and are mostly characterized by their propagules (spores, conidia, and sporangia) and are dispersed in or above the water. The described filamentous freshwater fungi are taxonomically more associated with the Ascomycota phylum, and only a small percentage is associated with Basidiomycota (Tsui *et al.* 2016) but comprise taxa from all fungal phyla.

Culture-based studies show that freshwater fungi dominate the microbial communities associated with plant litter in streams, in both woody and herbaceous debris (Hieber and Gessner 2002; Jabiol *et al.* 2019). They play a crucial role in carbon, nutrient, and energy flow in freshwater ecosystems due to their ability to secrete exoenzymes that catalyze the turnover of complex carbohydrate molecules such as cellulase, hemicellulase, and laccase, and other polysaccharides into smaller units, providing an energy source for the cell and organic matter for the aquatic food web, allowing the transfer of energy and nutrients to higher trophic levels (Bärlocher 1985; Gessner *et al.* 2007; Gulis *et al.* 2019; Graça *et al.* 2020).

Ecological—and in particular enzymatic—studies showed that freshwater fungi perform differently because they are equipped with different sets of enzymes (Suberkropp *et al.* 1983). The microbiological composition and extracellular enzymatic activity are also affected by an increase in temperature in freshwater environments (Fenoy *et al.* 2022), which can also be promoted by climate change (Seena *et al.* 2023) and likely prejudiced ecosystem functioning (Parain *et al.* 2019). On the other hand, fungal communities were functionally richer in the colder subregions (Fenoy *et al.* 2022) and are capable of decomposing leaf litter at lower temperatures (Taylor and Chauvet 2014; Pérez *et al.* 2018).

Many freshwater fungi are also equipped with special strategies to survive colder temperatures, and that can include the production of antifreeze proteins (AFPs) and cold-active enzymes, among other features. Those features contribute to fungi survival even in harsh conditions as found in arctic and subarctic streams (Brown 1978; Robinson 2001; Gessner and Robinson 2003; Hassan *et al.* 2016; Weinstein *et al.* 2019). The fungi AFPs in intracellular sites and extracellular site keep their cell fluids in the liquid state under cold conditions by inhibiting the formation of an ice crystal, resulting in a phenomenon called thermal hysteresis (Hoshino *et al.* 2009; Nath *et al.* 2013). This phenomenon enables the cell to endure freezing temperatures, and consequently, cell death (Hashim *et al.* 2013; Białkowska *et al.* 2020; Yusof *et al.* 2021). In temperate regions, the survival rate of aquatic fungi is less inhibited by water temperatures when near 0°C and can show growth peaks at temperatures between 15 and 25°C. The sporulation activity is also highest at lower temperatures than in most other fungi (Dang *et al.* 2009).

Genome sequence technology is an important tool to reveal the adaptation of organisms to specific environments and is shown to be an essential tool for our understanding regarding fungi diversity, mechanisms for plant cell wall degradation, and adaptation to cold environments (Morel *et al.* 2013; Rodrigues *et al.* 2019; Yusof *et al.* 2021).

The freshwater fungus *Filosporella fistucella* Marvanová and P.J. Fisher was first isolated and described in 1991 as an endophyte from alder roots (Dartmoor National Park, UK; Marvanová and Fisher 1991). Since then, this species has been reported in microbial ecology studies worldwide such as in India (Rajashekhar and Kaveriappa 2003), Germany (Carl *et al.* 2022), South Korea (Mun *et al.* 2019), and Portugal (Duarte *et al.* 2015). The strain *F. fistucella* CCM F-13091 is the ex-type of the species and was obtained from Ludmila Marvanová of the Czech Collection of Microorganisms (CCM). This species belongs to the Ascomycota class Leotiomycetes, order Helotiales, and is affiliated to Drepanopezizaceae (Baschien *et al.* 2013; Johnston *et al.* 2019).

## Materials and methods

### Isolation and identification

The *F. fistucella* culture CCM F-13091 was provided by the CCM and was maintained in the liquid nitrogen internal collection of DSMZ (Leibniz Institute DSMZ, German Collection of Microorganisms and Cell Cultures) under the number DSM 105177. For DNA extraction, the fungus was grown on 2% malt extract (Feelwell, Germany) agar (Oxoid, Germany) at 16°C in a cooling room. To confirm the identity of the *F*. *fistucella* culture, we applied Sanger sequencing of the internal transcribed spacer (ITS) and partial large subunit gene of the rDNA operon using primers ITS1F (5′-CTTGGTCATTTAGAGGAAGTAA-3′; Gardes and Bruns 1993) and LR5 (5′-TCCTGAGGGAAACTTCG-3′; Vilgalys and Hester 1990). The resulting sequences were manually curated using Sequencher tool v5.4.6 (http://www.genecodes.com), and then BLAST was performed against the National Center for Biotechnology Information (NCBI) nucleotide database (https://www.ncbi.nlm.nih.gov/). The ITS sequence is deposited under accession NR_153982 in the NCBI database.

### Cultivation and genome sequencing

After identity confirmation, the biomass of *F. fistucella* was transferred to a 1-L flask containing 500 mL of potato-glucose liquid medium (Carl Roth, Germany) using a sterile loop and kept in a shaker (Bottmingen, Switzerland) at 16°C and 120 RPM. Fourteen g of biomass of cells were used to extract genomic DNA (see Supplementary File 1) genome fragment sizes superior 15 kb were confirmed using Agilent Femto Pulse (Agilent, USA) and sent to Hifi genome sequencing at Macrogen, The Netherlands.

### Genome assembly and gene prediction

The CCS tool (https://ccs.how/) was used to generate the consensus reads from the raw sequencing data before assembly with Flye v2.9 (Freire *et al.* 2022) using the default parameters for PacBio Hifi assembly. The genome completeness was evaluated with BUSCO v5.3.0 (Simão *et al.* 2015) using the eukaryota_odb10 database.

Genome repetitive sequences were soft-masked using tantan (Frith 2011) before ab initio gene prediction with Funannotate v1.8.9 (Palmer and Stajich 2020, github.com/nextgenusfs/funannotate), using Augustus v3.3.3 (Stanke *et al.* 2006), SNAP (Korf 2004), glimmerHMM v3.0.4 (Majoros *et al.* 2003), and Genemarker-ES v4.6.2 (Ter-Hovhannisyan *et al.* 2008).

### Comparative genomics

The *F. fistucella* genome was compared against the freshwater fungus *Tricladium varicosporioides* (Tubaki) Johnston and Baschien (synonym: *Hymenoscyphus varicosporioides*) genome (Sivichai *et al.* 2003). The genome of *T. varicosporioides* was obtained from NCBI (accession number: GCA_021365295.1). The species *T. varicosporioides* was selected for the study because it is phylogenetically closely related to *F. fistucella* (Baschien *et al.* 2013; Johnston and Baschien 2020).

### Carbohydrate-active enzyme annotation and leaf-litter degradation enzymes

The genome annotation was performed in the dbCAN2 database (Zhang *et al.* 2018) using the probabilistic model of hidden Markov models (HMMer tool; Finn *et al.* 2011) to identify carbohydrate-active enzymes (CAZys; accessed on 2022 August 22) in both genomes. The analysis was performed against the dbCAN2 database for all 6 available classes of CAZys: carbohydrate-binding module (CBM), glycoside hydrolases (GHs), polysaccharide lyases (PLs), auxiliary activities (AAs), carbohydrate esterases (CEs), and glycosyl transferases (GTs).

The HMMer tool also provided the biochemistry function of the enzymes classified in the dbCAN2 by which were filtered for leaf-litter degrading enzymes (cellulase, hemicellulase, laccase, pectinase, cutinase, amylase, xylanase, and xyloglucanase; de Vries *et al.* 2017; Steindorff *et al.* 2021).

### Cold-adapt and antifreeze enzymes

The leaf-litter enzymes identified were submitted to the random forest algorithm of the CryoProtect webtool (Pratiwi *et al.* 2017) to predict whether the identified CAZy sequences associated with plant-litter degradation could have antifreeze properties.

### Secondary metabolites

Biosynthetic gene clusters (BGCs) were predicted using the unannotated genome assembly from the Flye assembler in the Antismash v6.1.1 fungal version. The detection strictness was set to relax, as default. To detect secondary metabolites in fungal Antismash, all extra feature options were activated, including the CASSIS tool (Baudet *et al.* 2010).

## Results

### Genome features

The *F. fistucella* genome assembly (45.7 Mb) has a smaller size than *T. varicosporioides* (48.2 Mb) but with a higher N50 value. Despite that completeness and BUSCO fragments present similar values between both genomes, as shown in Table 1.

**Table 1.** Genome size, quality, and completeness comparison of *F*. *fistucella* and *T*. *varicosporioides* genomes.

BUSCO data	*F. fistucella*	*T. varicosporioides*
Genome size	45.7 Mb	48.2 Mb
No. of contigs/scaffolds	21	106
N50	2.6 Mb	1.1 Mb
L50	6	15
GC content	45.31%	44.58%
Completeness	C: 98.4% (S:98.4%, D:0.0%)	C: 98.4% (S:97.6%, D:0.8%)
Fragments	1.2% (3 fragmented BUSCO)	F: 1.2% (3 fragmented BUSCO)
Missing	0.4% (1 missing BUSCO)	0.4% (1 missing BUSCO)
Max. length of scaffold	6,242.644	2,716.377
Mean sequence length	2,179,724	455,114
CDS	13,507	12,628

### Gene prediction and annotation with CAZys

The ab initio gene prediction with Funannotate identified 12,628 proteins in *T. varicosporioides*, and 13,507 proteins for *F. fistucella*. We have analyzed all predicted genes against all 6 classes of CAZy available in dbCAN2: CBM, GHs, PLs, AAs, CEs, and GTs, as shown in Table 2. The *F*. *fistucella* genome has more plant-litter degradation enzymes than *T*. *varicosporioides.* Respectively, we annotated 855 and 719 proteins in the dbCAN2 database for CAZys.

**Table 2.** Number of CAZys matching the dbCAN2 database using the HMMer tool for the 6 classes of enzymes available on the database for *F. fistucella* and *T. varicosporioides*.

CAZy classes	*F. fistucella*	*T. varicosporioides*
Glycoside hydrolases (GH)	419	342
Auxiliary activities (AA)	158	154
Glycosyl transferases (GT)	122	104
Carbohydrate esterases (CE)	70	64
Carbohydrate-binding module (CBM)	62	43
Polysaccharide lyases (PL)	24	12

### Plant-litter degradation enzymes

The results of CAZys annotation were filtered for the enzymes associated with the degradation of different plant-based polysaccharides (cellulose, xylan, xyloglucan, starch, hemicellulose, lignin, pectin, and cutin). The *F. fistucella* genome presented a higher number of enzymes associated with leaf-litter degradation than *T. varicosporioides*, 215 and 178, respectively, as shown in Table 3.

**Table 3.** Differences in numbers of CAZys annotated with the dbCAN2 database in the genomes of *F*. *fistucella* and *T*. *varicosporioides* and the enzymes associated with plant leaf degradation.

	*F. fistucella*		*T. varicosporioides*	
	Total	Antifreeze	Total	Antifreeze
Cellulose	44	17	44	18
Xylan	58	21	53	19
Xyloglucan	12	3	10	4
Starch	19	6	21	8
Hemicellulose	5	3	3	1
Lignin	35	15	28	7
Pectin	37	9	32	9
Cutin	5	1	2	0

### Antifreeze properties

The sequences classified as plant-litter degrading enzymes were analyzed with Cryoprotect, where we identified 75 enzymes in *F. fistucella* and 66 enzymes in *T. varicosporioides* predicted as AFPs, as shown in Table 3.

### Secondary metabolites

Using secondary metabolites analysis with Antismash, we identified a cluster-type terpene in *F. fistucella* with 40% similarity with squalestatin S1 (also found in *T. varicosporioides* with 40% similarity), a common fungi metabolite (Lebe and Cox 2019). In addition, 13 nonribosomal peptide synthetase (NRPS) clusters and 14 polyketide synthasis (T1PKS) clusters were found, mostly with unknown metabolic functions or with low similarity with known fungi metabolites, such as eupenifeldin (Bunyapaiboonsri *et al.* 2008; Zhai *et al.* 2019) and naphthalene (Smith *et al.* 1976; Cerniglia *et al.* 1978; Hofmann 1986; Sutherland 1992).

We identified fewer BGCs in *T. varicosporioides* than in *F. fistucella*. In *T. varicosporioides*, we identified 6 T1PKS (PKS type 1), 7 NRPS, 3 hybrid clusters PKS-NRPS, and 1 hybrid cluster terpene-PKS. In *F*. *fistucella*, we identified 11 T1PKS (PKS type 1), 10 NRPS, 3 hybrid clusters PKS-NRPS, and 1 terpene cluster. Most BGCs identified have unknown or low similarities within the Antismash secondary metabolites database. The clusters with high similarities were observed on 2 occasions in each organism. The antimicrobial compound pyranonigrin E was predicted in both genomes with 100% similarity to the sequence of *Aspergillus niger* within the Antismash database, and it is the only metabolite predicted in both *F. fistucella* and *T. varicosporioides*. Apart from this, unique metabolites with high similarity within the database were identified in both genomes. The *F. fistucella* had 100% similarity to the 6-methylsalicylic acid. The *T. varicosporioides* had 100% similarity with fusarin.

## Discussion

The genome size of the *T. varicosporioides* (48.2 Mb) and the newly assembled *F. fistucella* (45.7 Mb; Table 1) are slightly similar to previous aquatic fungi genomes described: *Margaritispora aquatica* (42.5 Mb; Goh, Mun, Park, *et al.* 2019), *Hymenoscyphus tetracladius* (current name *Articulospora tetracladia*; 41.8 Mb; Goh *et al.* 2018), *Lepidopterella palustris* (46 Mb; Peter *et al.* 2016), *Tetracladium marchalianum* (Anderson and Marvanová 2020), *Aquanectria penicillioides* (53.7 Mb; Goh, Mun, Oh, *et al.* 2019), and *Clavariopsis aquatica* (34.1 Mb; Heeger *et al.* 2021). We were also able to acquire a genome with higher quality than the phylogenetically closely related genome of *T. varicosporioides.* Both genomes present a similar level of completeness and quality, although *T. varicosporioides* genome has an inferior quality to *F. fistucella* (Table 1).

Although having a smaller genome size than *T. varicosporioides*, we detected more genes through ab initio prediction in *F. fistucella*. A vast number of genes encoding CAZys were identified in both freshwater fungi (855 in *F. fistucella* and 719 in *T. varicosporioides*).

Aquatic fungi are major decomposers of plant litter in aquatic environments and can use a wide range of carbohydrates (such as starch, cellulose, cellobiose, sucrose, mannose, xylose, maltose, glucose, and galactose), through the breakdown of cell polysaccharides present in the cell wall and inside the cell (Abdel-Raheem and Ali 2004; Sati and Bisht 2017; Bärlocher *et al.* 2020). These cell components can be divided into 3 groups: (1) water-soluble amino acids and sugars, (2) the polysaccharides hemicellulose, cellulose, cutin, and pectin, (3) lignin and other aromatic compounds (Fioretto *et al.* 2005; Bani *et al.* 2018). The polysaccharides can be reduced into monosaccharides through enzymatical activities by fungi and be used for energy or as precursors for the biosynthesis of biomolecules (Wang *et al.* 2020).

Fungi that use plant tissue as a carbon source acquired a large diversity of plant cell wall-degrading or wall-modifying enzymes (Hage *et al.* 2021). There are 5 different CAZy classes associated with the breakdown of carbohydrates, namely, GHs, PLs, AAs, CEs, and GTs. These enzymes provide control of chemical reactions for the breakdown of oligo- and polysaccharides (Lombard *et al.* 2014). In our analysis, *F*. *fistucella* had more CAZys in all 6 categories of enzymes in the dbCAN2 database, which show a higher capacity to degrade plant cell wall and their components than *T*. *varicosporioides*.

Pectin is an important class of plant cell wall polysaccharides. It is located in the middle lamella of the leaf and constitutes of D-galacturonic acid as the main component (Broxterman and Schols 2018; Marić *et al.* 2018). Pectin is reported to account for up to 20–25% of the total dry weight in some plants (Schmitz *et al.* 2019). Lignin is a complex aromatic heteropolymer and an important organic component of plant litter, accounting for ∼5–35% of leaf-litter dry mass. Despite being so abundant in the cell, they are slowly decomposed because of their structural properties. Their degradation is an important process because lignin protects most of the cellulose and hemicellulose from enzymatic hydrolysis (Boerjan *et al.* 2003; Jabiol *et al.* 2019).

Cellulose is the most abundant polysaccharides component in plants and one of the most abundant polysaccharides in the world (Chundawat *et al.* 2011; Dadwal *et al.* 2021). The breakdown of cellulose is made through cellulase production, which may be enhanced by external hemicellulose (Wang *et al.* 2020). Hemicelluloses are diverse and complex polysaccharides that are classified according to the main sugar in the backbone of the polymer, i.e. xyloglucan, xylan, galacto(gluco)mannan (Andlar *et al.* 2018; Wang *et al.* 2020). Among them, the most abundant hemicellulose is xylan (Moreira and Filho 2016), by which the fungi can degrade as their primary carbon source (Collins *et al.* 2005).

Cutin shares a complex barrier together with other biopolymers, such as suberin and sporopollenin, and is involved in waterproofing the leaves and fruits of higher plants (Heredia 2003; Domínguez *et al.* 2015). Starch is a major storage of polysaccharide in plants and is degraded by α-amylase and β-amylase (Papa *et al.* 2008) being good sources of carbon for some species of aquatic fungi since it is more easily degraded than most other cellular components (Abdullah and Taj-Aldeen 1989; Baudy *et al.* 2021).

In our study, *F. fistucella* has a richer set of enzymes capable of degrading plant biomass than *T. varicosporioides* with the exception of cellulose (44 in both organisms) and starch degradation (19 and 21, respectively), and more enzymes for xylan, xyloglucan, hemicellulose, lignin, pectin, and cutin. *Filosporella fistucella* has shown to have a more complex enzymatic system to act in leaf-litter degradation by triggering enzymes involved in leaf and wood cell wall degradation, as well as sugar molecules. These enzymes participate in the assembly of carbohydrates, which suggests a higher potential for growth in the water, and a more broad adaptation to the freshwater environment.

The *F. fistucella* and *T. varicosporioides* genomes had a similar number of AFPs, showing a similar capacity to produce CAZys that can help the cell to survive in cold conditions and keep microbial decomposition of leaf litter. AFPs are a vital mechanism for adaptation in freshwater. Low freshwater temperatures (below 4°C) may cause organelle damage and cell death by severely deteriorating an organism’s cell membranes (Baskaran *et al.* 2021). Therefore, in order to survive in cold environments, freshwater fungi can produce AFPs as one of the survival strategies, protecting cell membranes and their structural integrity (Ustun and Turhan 2015).

Freshwater fungi provide essential ecosystem services in aquatic systems (Seena *et al.* 2023). Besides their key functional adaptations to leaf material degradation, they promise to be potent sources of compounds such as peptides, polyketides, terpenes, and alkaloids, although, very much of their biology and chemistry are still to be known (Hernández-Carlos and Gamboa-Angulo 2011; Bills and Gloer 2017; El-Elimat *et al.* 2021). In our study, many BGCs are not yet identified or had low similarities in both organisms' genomes against the Antishmash database. This can be indicative that an important group of microorganisms is worth to be further studied which could potentially have a vast number of commercial and scientific applications (Goh and Hyde 1996; Hernández-Carlos and Gamboa-Angulo 2011; Hassan *et al.* 2022).

Microbial natural products from freshwater aquatic fungi present exciting opportunities to discover natural alternatives for commercial antimicrobial products (El-Elimat *et al.* 2021; Canto *et al.* 2022). Many studies identified antimicrobial compounds in several aquatic fungi (Poch *et al.* 1992; Harrigan *et al.* 1995; Oh *et al.* 1999, 2001, 2003; Mudur *et al.* 2006; Gloer 2007). The secondary metabolite pyranonigrin E is an antimicrobial compound found in both aquatic fungi genomes approached in this study. The pyranonigrin E compound is a PKS-NRPS hybrid metabolite and has a considerable interest as a potent antioxidant (Awakawa *et al.* 2013).

Many ascomycete fungi produce squalestatins (Bills *et al.* 1994). The squalestatins, identified in both genomes, can inhibit the first step of cholesterol biosynthesis by targeting squalene synthase (Sidebottom *et al.* 1992). Due to this characteristic, they have antifungal properties utilized in the development of medicine (Baxter *et al.* 1992; Sidebottom *et al.* 1992; Bergstrom *et al.* 1995).

New microbial compounds are being discovered mainly through metabolomics and chemistry studies with a variety of structural and metabolic capacities and are the basis for bioinformatics predictions. Despite the large use of bioinformatics tools such as antiSMASH, SMURF (Khaldi *et al.* 2010), or CASSIS/SMIPS (Wolf *et al.* 2016) for BGC predictions, they are still far from perfect due to limitations in the recognition of metabolites outside the clusters PKS and NRPS. Additionally, clusters that are dispersed across multiple chromosomes and that are simultaneously co-regulated have a chance of being missed by the algorithms (Chavali and Rhee 2018). Improvement in secondary metabolite isolations and/or metabolomic studies, coupled with genome sequencing annotation for BGCs, could help to shed light on the uniqueness of secondary metabolite biosynthesis in freshwater fungi (El-Elimat *et al.* 2021).

Based on the results, *F. fistucella* has a higher capacity for adaptation to the freshwater environment, because of its richness in CAZys and AFPs, can indicate that it (1) may be more competitive and (2) may have a higher biotechnological potential than *T. varicosporioides* (Kumar *et al.* 2021; Kumari *et al.* 2021; Palaniappan *et al.* 2021).

## Supplementary Material

jkad190_Supplementary_Data

## Data Availability

The *F. fistucella* genome assembly presented in this manuscript is submitted to the National Center for Biotechnology Information (NCBI) under accession number GCA_030077755.1. The raw reads and genome assembly can be found at NCBI with Bioproject number PRJNA924252. The CAZy Genome annotations are available at GitHub (https://github.com/rissidaniel/Ffistucella_cazy). Supplemental material available at G3 online.
