# Supplementary material for: Comparative genome analysis of the freshwater fungus Filosporella fistucella indicates potential for plant-litter degradation at cold temperatures
Source: G3 (Bethesda). 2023 Aug 24;13(11):jkad190. doi: 10.1093/g3journal/jkad190 (PMC10627260; doi:10.1093/g3journal/jkad190)
Supplement: jkad190_Supplementary_Data [file jkad190_supplementary_data.docx]

**S1: Supplementary Material:**

**Comparative genome analysis of the freshwater fungus *Filosporella fistucella* indicates potential for plant litter degradation at cold temperatures**

Daniel Vasconcelos Rissi; Maham Ijaz; Christiane Baschien*

**Genome DNA extraction for Genomics Analysis**

**Preparatory steps**

Prepare 1000 ml Erlenmeyer flask with sterile 500 ml of 20% potato extract glucose broth. Inoculate with a block (approx. 1*1 cm) of *F. fistucella*-colonized agar. For four weeks, incubate the strain at 16 °C under 100 rpm of shaking until sufficient mycelium (about 14 g of biomass) has developed without spore formation. Transfer the biomass to a 50 ml falcon tube avoiding transfer of the medium and centrifuge it at 800 rpm for 5 minutes. Discard the supernatant containing media and wash the mycelia with 30 ml sterile distilled water. Centrifuge again and repeat the process until broth clean biomass is obtained.

**Chilling and grinding**

Store the biomass at -20°C for at least 24hrs. Freeze dry the biomass. In Our study, the biomass was freeze-dried using an Alpha 1-4 LD unit (Christ®, Germany). Add 2 sterile glass spheres 3 mm and place in a beat mixer (MP FastPrep-24^TM^) until the mycelium is crushed (5.5 m/s;30 s; 2x50 ml).

**Protein Extraction**

Add 20 ml CTAB buffer (Serva®, Germany) (in the fume hood) mix, and incubate for 30 minutes at 65°C**.** Shake briefly every 10 minutes by hand**.** Allow cooling for five minutes on the ice. Add 20 ml of Chloroform Isoamyl [24:1] (Serva®, Germany) and gently invert for 1 minute**.** Centrifuge for 10 minutes at ~3200 rpm. Transfer the supernatant to a new tube and add an equal volume of Isopropanol (J.T. Baker®, Poland)**.** Gently Mix by inverting the tube (Optionally can be left overnight at -20°C)**.**

**RNase treatment and DNA Isolation.**

Centrifuge for 10 min at ~3200 rpm. Carefully remove the supernatant and wash the pellet with 5 ml ice-cold 70% EtOH. Remove the ethanol by centrifuging for 10 minutes at 3200 rpm at 4 °C. After the pellet has been air-dried for 15 to 20 minutes, resuspend it in 100 to 200 µl of 1X TE buffer. Dilute the DNA in 100 µl of 1x TE buffer in a shaker at 50 °C for 1 hr. Add 15 µl of RNAse, then stir for an hour at 37 °C. Genomic DNA-containing tubes should be kept at -20 °C for short time storage.
